# TagDust2: a generic method to extract reads from sequencing data

**DOI:** 10.1186/s12859-015-0454-y

**Published:** 2015-01-28

**Authors:** Timo Lassmann

**Affiliations:** 1 RIKEN Center for Life Science Technologies (CLST), RIKEN Yokohama Institute, 1-7-22 Suehiro-cho, Tsurumi-ku, Yokohama, 230-0045 Kanagawa Japan; 2 Telethon Kids Institute, The University of Western Australia, 100 Roberts Road, Subiaco, Subiaco, 6008 Western Australia Australia

**Keywords:** Next generation sequencing, TagDust

## Abstract

**Background:**

Arguably the most basic step in the analysis of next generation sequencing data (NGS) involves the extraction of mappable reads from the raw reads produced by sequencing instruments. The presence of barcodes, adaptors and artifacts subject to sequencing errors makes this step non-trivial.

**Results:**

Here I present TagDust2, a generic approach utilizing a library of hidden Markov models (HMM) to accurately extract reads from a wide array of possible read architectures. TagDust2 extracts more reads of higher quality compared to other approaches. Processing of multiplexed single, paired end and libraries containing unique molecular identifiers is fully supported. Two additional post processing steps are included to exclude known contaminants and filter out low complexity sequences. Finally, TagDust2 can automatically detect the library type of sequenced data from a predefined selection.

**Conclusion:**

Taken together TagDust2 is a feature rich, flexible and adaptive solution to go from raw to mappable NGS reads in a single step. The ability to recognize and record the contents of raw reads will help to automate and demystify the initial, and often poorly documented, steps in NGS data analysis pipelines. TagDust2 is freely available at: http://tagdust.sourceforge.net.

**Electronic supplementary material:**

The online version of this article (doi:10.1186/s12859-015-0454-y) contains supplementary material, which is available to authorized users.

## Background

Next generation sequencing has greatly accelerated the accumulation of genomics data. Different protocols targeting the genome, epigenome and transcriptions are widely used [1]. In essence, all protocols capture biological sequences of interest while adding adaptors and other sequences to facilitate cost effective sequencing. An obvious examples is the use of indices or barcodes allowing researchers to multiplex sequencing experiments [2,3]. In addition recent protocols include random oligos in the library construction to correct for PCR and other biases [4]. As the length of such auxiliary sequences increases so is the chance that sequencing errors occur in these key sequences. In the best case these errors lead to some sequences being lost to the downstream analysis, but in the worse case sequences can be mixed up between samples leading to analytical noise. A compounding complication is that error rates of current sequencing instruments vary and is not obvious how to select an appropriate strategy to process the raw data.

Programs have been developed to tackle individual steps in this general area including the removal of artefacts from raw NGS sequencing files [5], read trimming [6,7] and read demultiplexing [8-10]. All programs are based on matching known strings to the sequenced reads. However none tackle the whole problem comprehensively necessitating the construction of processing pipelines [11-13] to obtain clean sequences for assembly or mapping to a reference sequence. Parameterization and maintenance of these pipelines for individual NGS assays is cumbersome and only exacerbated by the rapid pace of protocol development [14].

**Figure 1:**
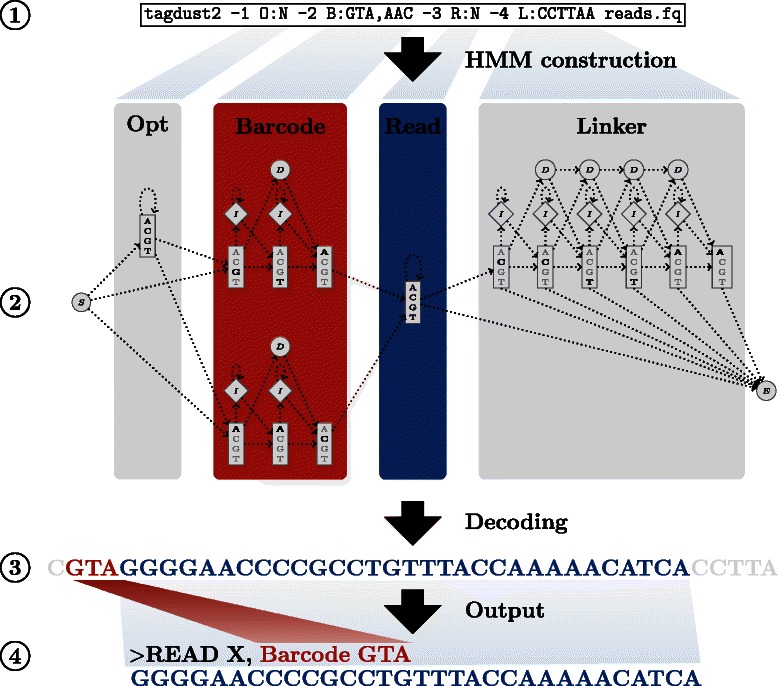
**Overview of the TagDust2 workflow.** **1)** A user specifies the expected read architecture as a sequence of pre-defined blocks. Here there are four of such blocks. **2)** A HMM is constructed by concatenating the pre-defined blocks in the order given by the -1 …command line options. For example -2 B:GTA,AAC is translated into the second (red) part of the HMM and models the presence of two mutually exclusive barcode sequences. **3)** Reads are scanned with the HMM and each nucleotide is labelled by the block it belongs to. In the example shown the three letter barcode GTA is recognised in the raw sequence. **4)** Based on the labelling of the sequence, a barcode is assigned to each read and remaining sequences are trimmed.

TagDust2 solves this problem in a generic way while guaranteeing good accuracy. The key part of finding the mappable read and matching it to a barcode is carried out by a user defined hidden Markov model (HMM). This approach has several key advantages. Firstly, a user defined architecture gives great flexibility in respect to the different sequencing assays used. Secondly, HMMs themselves are well suited to recognize sequences even in the presence of sequencing errors. Artifacts such as primer dimers will by definition not match the HMM and are therefore easily recognized and discarded (see Figure 1). Finally, by comparing reads to multiple HMMs it is trivial to detect the library protocol used. The latter can be used in production environments to automatically recognize and process different NGS protocols.

## Approach

Let us define the read architecture as the order of adapters, barcodes and the mappable sequence itself. TagDust2 contains a library of HMM models, each designed to capture the different types of sequences one might encounter in real raw NGS reads. For example, one model recognizes partial sequences such as 5’ and 3’ adapters while another captures the presence of mutually exclusive barcode sequences used for multiplexing libraries.

Each sequenced read is scored against the global HMM using the forward algorithm [15] and a zero order background model. If barcodes are present in the architecture an additional background HMM of the same length as the barcode is introduced. The backward algorithm is then used to determine the total probability of the most likely barcode sequence used. All probabilities are converted into a phred scaled extraction quality analogous to the mapping quality introduced in MAQ [16]. Simply put, the extraction quality reflects how certain we can be that the read matches the read architecture and a particular given barcode. To finally extract the read sequence TagDust2 employs an optimal accuracy decoder [17].

In transcriptome sequencing it is common to exclude ribosomal RNAs from the downstream analysis. In addition users may wish to extract known sequences such as spike-ins to perform separate analysis. For completeness TagDust2 includes the option to scan all extracted reads against a database of known contaminants and exclude them from mapping. Similarly, a low-complexity filter is included. Taken together TagDust2 performs all steps required to go from raw to mappable sequences and therefore greatly simplifies processing pipelines.

## Implementation

TagDust2 is a complete departure from the original TagDust [5] program. TagDust2 implements a small library of HMMs referred to as segments. Each segment contains a silent start and end state which are used to connect multiple segments. Segments are hand-designed to capture commonly occurring features in raw sequences such as the combination of barcodes, variable length sequences and so on (see the user manual for a complete list). Users can use a simple command line interface to specify the expected sequence of segments in their reads. TagDust2 automatically constructs a global HMMs from the segments and starts scoring the individual reads (see Figure 1). Alternatively users can create a file containing a selection of pre-defined architectures. TagDust will score reads against all architectures and determine the most appropriate match.

Internally, TagDust2 uses a full profile HMMs for each segment. To emulate different segments I simply set some transition probabilities to zero. For example the HMM segment corresponding to the actual read is implemented as a profile HMM with one column and transitions directly to and from the insertion state. The most computationally demanding parts are parallelized using threads. TagDust2 is written in C, extensively documented and the source code is freely available.

### Sequence scoring

In short read mapping the mapping quality *Q* reflects the confidence we have in one particular mapping location over all others [16]. Analogously, TagDust2 compares the probability of each read matching to the user specified HMM to the total summed probability including a random model:

$$P = 1 - \frac{P(x|M)^{\ast} V}{P(x|M) + P(x|R)} $$

where *P*(*x*|*M*) is the total summed probability of a read matching the model derived by the forward algorithm and *P*(*x*|*R*) is the probability of the read give a random zero order Markov model. *V* represents the fraction of *P*(*x*|*M*) corresponding to the most likely barcode sequence, if present or 1 otherwise. It is defined as the most probable transition from a silent state *s* to the first match state *m* of a barcode *j*:

$$V = \max_{j} \left(\frac{f_{s}(i) a_{s,m_{j}} e_{m_{j}}(x+1) b_{m_{j}}(i+1)}{\sum\limits_{\pi} P(x,\pi | M)}\right) $$

where *f*_*s*_ is the total probability summed up over all paths *π* leading up to state *s* and $b_{m_{j}}$ the total probability of all paths starting from *m*_*j*_. *a* and *e* are the transition and emission probabilities respectively. The denominator is the total probability of the sequence *x* given the model *M* summed over all paths *π*.

To further reduce possibility of one barcode being mixed up with one another TagDust2 always adds one additional HMM to the segment. The latter is of the same length as other barcodes but the emission probabilities are set to the background frequencies. This model captures raw sequences in which the barcode sequence is too ambiguous to be uniquely assigned.

### Threshold determination

Selecting an appropriate threshold on *P* is not trivial. A stringent threshold is appropriate when many auxiliary sequences are present. However, the same threshold may yield no results when simple read architectures are given because the distributions of *P*(*x*|*M*) and *P*(*x*|*R*) will overlap. In other words thresholds should be set independently for different read architectures.

To set thresholds dynamically and automatically, TagDust2 simulates, or emits, reads from the model *M* and the random model *R*. All reads are scored and the threshold is set to the value that gives the best sensitivity plus specificity.

### Optimal accuracy decoding

To obtain the most probable labeling of a raw sequence, TagDust2 employs the optimal accuracy decoding algorithm as described in [17]. To apply this algorithm to our problem the label probability of a nucleotide is defined by the summed posterior label probabilities of all states belonging to a particular HMM segment. A secondary dynamic programming algorithm is used to determine the path with the maximum posterior label probability, constrained by the global HMM architecture. The label probabilities are essentially used as a substitution matrix while the architecture is enforced by the equivalent of gap penalties.

If fingerprints, a random sequence added to detect PCR artifacts, are present TagDust2 checks at this stage if the length after decoding matches the users input. If not the read is discarded.

### Further read filtering

TagDust2 allows users to specify a fasta file containing known sequences the user wishes to exclude from mapping. For example in transcriptome sequencing one commonly wants to remove ribosomal sequences from the downstream analysis. To address this issue in a general manner TagDust2 exhaustively align all reads to the target reference sequences and discard all reads with less than a user defined number of mismatches, insertions and deletions. For efficiency I implemented the Myers bit parallel algorithm [18] using SIMD instructions as well as using thread level parallelism. The number of reads matching different reference sequences are automatically recorded.

### Filtering low complexity sequences

Tagdust2 implements a simplified version of the DUST module (R. Tatusov and D.J. Lipman, unpublished data) to filter out low complexity reads. The algorithm is only applied to the first sixty-four nucleotides of the reads.

## Results

To assess the performance of TagDust2 I generated large datasets by varying the number of barcodes used, their lengths and the per base error rate. In all experiments 90 thousand sequences containing the expected architecture were generated. An additional 10 thousand random sequences were added to assess the number of false positives. To obtain sets of barcodes with maximal dissimilarity I used programs described by Faircloth et al. [19]. Given that the original read and sample are known the number of reads assigned to the wrong sample and the total number of extracted reads can be quantified.

I compared the performance of TagDust2 to cutadapt [7], the fastx-toolkit [10] and Btrim [8] using default parameters.

The TagDust2 software distribution contains a document written using the literate programming paradigm to reproduce all the results presented here (see also Additional file 1).

### Basic de-multlexing

**Figure 2:**
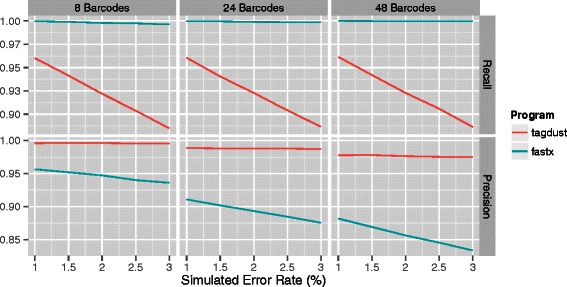
**Demultiplexing of libraries with 4nt barcodes assuming different error rates.** From left to right: simulations using 8, 24 or 48 different barcodes. The top panels show the recall and the bottom panels the precision. TagDust sacrifices recall for high precision.

A simple application of TagDust2 is de-multiplexing of libraries. In two separate experiments, four and six nucleotide long barcodes were appended to reads. The number of barcodes and their length was varied together with the sequencer error rate. The maximum similarity between any pair of barcodes was two when using 4nt and three when using 6nt barcodes (Figure 2).

TagDust2 is more conservative at extracting reads compared to fastx when using 4nt barcodes. As the number of barcodes and error rates are increased the precision of both programs is decreasing. However, the precision of TagDust2 is consistently higher compared to fastx and far less affected by the per-base error rate.

**Figure 3:**
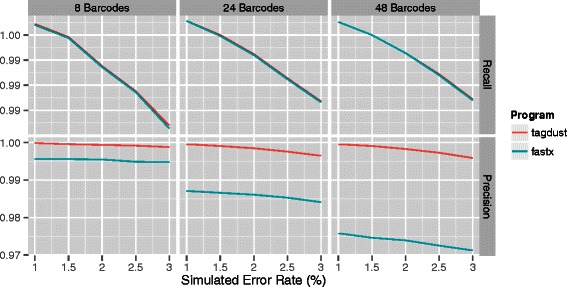
**Demultiplexing of libraries with 6nt barcodes assuming different sequencer error rates.** From left to right: simulations using 8, 24 or 48 different barcodes. The top panels show the recall and the bottom panels the precision.

Increasing the barcode length to six nucleotides makes it much easier to unambiguously assign reads to a particular sample (Figure 3). Here there is no appreciable difference in recall between the two programs. As before, TagDust2 is consistently more precise compared to fastx.

### De-multiplexing in the presence of 5’ and 3’ adapters

**Figure 4:**
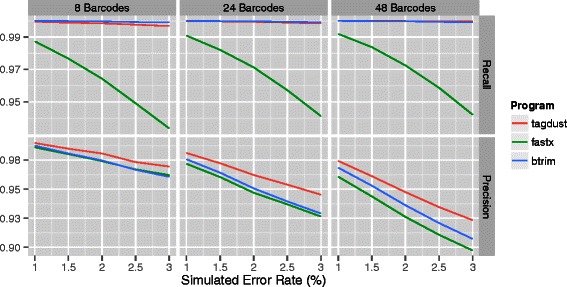
**Demultiplexing of libraries with 5‘ and 3‘ linkers and 4nt barcodes assuming different sequencer error rates.** From left to right: simulations using 8, 24 or 48 different barcodes. The top panels show the recall and the bottom panels the precision.

**Figure 5:**
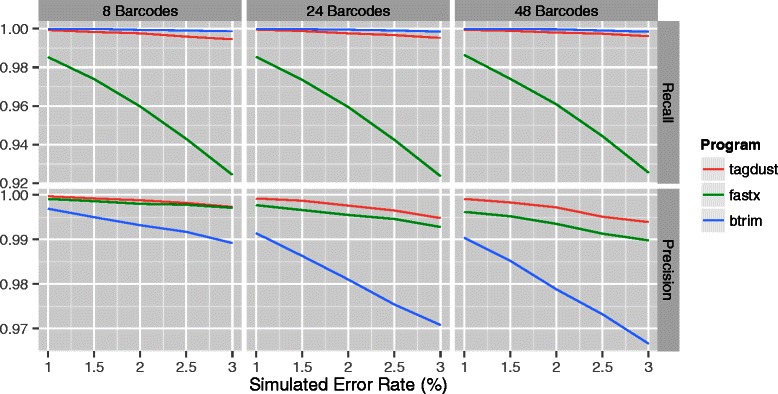
**Demultiplexing of libraries with 5‘ and 3‘ linkers and 6nt barcodes assuming different sequencer error rates.** From left to right: simulations using 8, 24 or 48 different barcodes. The top panels show the recall and the bottom panels the precision.

In a more complicated case, we add both 5’ and 3’ adapters (AGGGAGGACGATGCGG and GTGTCAGTCACTTCCAGCGG) to the simulated case from before (Figures 4 and 5). TagDust2 performs favorably in these cases. The additional long sequences make it easy to differentiate between real and random sequences and hence the recall is high.

Perhaps most importantly, the syntax for running TagDust2 is virtually unchanged from before. Working with more complex architectures is simple, negating the need to write many protocol specific scripts for data processing.

### Automatic detection of architectures

In parallel to running TagDust2 with a case specific command line for each of the situations above, I also ran TagDust using a pre-specified architecture file. In brief, this file contains a list of all possible architectures used in all the examples above. In all cases, TagDust2 run in this mode selected the correct architecture out of all the other options and produced identical results to those shown in Figures 2, 3, 4 and 5. In other words, TagDust2 using the same options could recognize the presence or absence of adapters, distinguish between different numbers of 4nt or 6nt barcodes while being able to give good results irrespective of the sequencing error rate.

### Comparison to the Casava pipeline

On Illumina sequencing instruments de-multiplexing of samples is performed automatically by the program bcl2fastq as part of the CASAVA pipeline. This program simultaneously converts the per-cycle BCL basecall files to per-read fastq files. BCL files are not readily available but fortuitously, the bcl2fastq software [9] is distributed together with a validation dataset containing the raw files of one lane of multiplexed paired-end samples (RUN 110120_P20_0993_A805CKABXX). The lane contains two human samples and a PhiX control sample indexed by 6nt long sequences (ACAGTG, ACTTGA, TTAGGC). By turning off the de-multiplexing functionality in bcl2fastq I could perform the de-multiplexing separately with TagDust2. All reads were aligned to their matching reference genomes (GRCh38, NC_001422.1) using BWA-MEM [20].

TagDust2 was able to assign more reads to all of the three samples compared the files produced by bcl2fastq (Table 1). Mapping the reads to their references demonstrates that the additional reads extracted by TagDust2 are usable for downstream analysis.

**Table 1:** **Comparison to Illumina’s bcl2fastq**

	**Sample**	**bcl2fastq**	**TagDust2**	**Difference (TagDust2 -**
				**bcl2fastq)**
Number of	AR005	1.472.734	1.508.848	36.114
extracted reads				
	AR008	1.518.208	1.543.918	25.710
	PhiX	49.104	51.488	2.384
Number of	AR005	1.435.567	1.464.037	28.470
aligned reads				
	AR008	1.479.158	1.498.524	19.366
	PhiX	46.369	48.296	1.927

### Real datasets

To highlight the performance of TagDust2 on real datasets with complicated read architectures I obtained the raw reads from a single cell transcriptome dataset [21] using 96 barcodes directly from the authors and a dataset containing unique molecular identifiers [4] (European nucleotide archive accession numbers: ERR048988 - ERR048994).

The raw reads from the single cell transcriptome dataset contain a 6 nucleotide barcode sequence followed by three guanines and then the actual read sequence. TagDust2 extracted 18.6 million additional reads compared to the original data processing pipeline used (Table 2). After mapping to the mouse genome using Tophat2 [22], 3.5 million additional reads could be aligned.

**Table 2:** **Summary of single cell extracted reads**

**Description**	**Original**	**Using TagDust2**
Total reads	110.622.138 (100.00%)	110.622.138 (100.00%)
Extracted	82.325.179 (74.42%)	100.900.895 (91.21%)
Mapped	66.204.974 (59.85%)	69.685.999 (62.99%)

**Figure 6:**
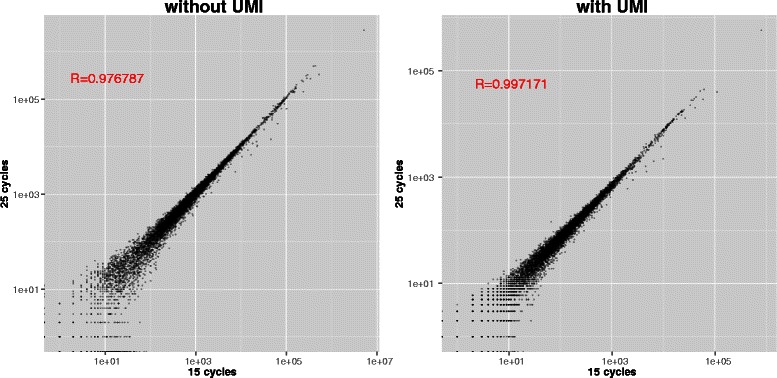
**Sample to sample correlation can be improved by using unique molecular identifiers.** The right panel shows correlation between samples using 15 and 25 PCR cycles without using the UMI sequences. The left panel shows the same data after collapsing all reads mapping to the location and containing the same UMI. TagDust correctly identified PCR artifacts based on their UMI sequences.



In the second case each read contains a random 10 nucleotide unique molecular identifier (UMI). Finding the same UMI associated with reads mapping to same location is a good indicator that these reads are PCR duplicates. TagDust2 automatically recognizes UMI sequences and converts them into a unique number. To understand whether the UMIs actually help in reducing technical noise caused by PCR amplification I compared libraries amplified using either 15 or 25 PCR cycles. After collapsing reads mapping to the same region with the same UMI the sample to sample correlation could be improved (Figure 6). More importantly, TagDust2 was able to extract the reads using the short command line:

tagdust2 -1 F:NNN -2 S:T -3 F:NNNN -4 S:T -5 F:NNN -6 B:GACTT -7 S:GGGG -8 R:N

specifying that we expect a 10 nucleotide fingerprint sequence (F:) separated by thymidines (S:) followed by a 5 nucleotide barcode (B:), 4 guanosines and finally the mappable read (R:). Apart form illustrating the inherent flexibility of TagDust2 when using complicated architectures, the command line itself also documents the contents of the raw sequences. Such information completely demystifies the early steps in NGS processing pipelines.

## Conclusions

The experiments on simulated and real data indicate that TagDust2 improves the initial steps in read processing in several ways. Firstly, using HMMs allows TagDust2 to effectively work datasets with a broad range of sequencing error rates. Secondly, the implementation of a library of possible read segments makes TagDust2 very accessible and useable. Finally, the automatic selection of architectures and thresholds greatly simplifies and generalizes the initial parts of data processing pipelines. In production environments TagDust2 allows users to define several read architectures and use the same pipeline for the pre-processing of diverse data types.

The approach presented here allows researchers to extract reads accurately and with a performance guarantee. Together with the two additional post processing functions, TagDust2 is a one stop solution to go from raw to mappable reads. The inherent flexible design makes TagDust2 applicable to a wide spectrum of current and future datasets.

## Availability and requirements

**Project name:** TagDust**Project home page:** http://tagdust.sourceforge.net **Operating systems:** Unix/Linux**Programming language:** C**Other requirements:** NA**License:** GNU General Public License version 3.0 (GPLv3)

## Additional file

Additional file 1
**Code and description to reproduce the figures in the manuscript.**
