# Supplementary material for: TagDust2: a generic method to extract reads from sequencing data
Source: BMC Bioinformatics. 2015 Jan 28;16:24. doi: 10.1186/s12859-015-0454-y (PMC4384298; doi:10.1186/s12859-015-0454-y)
Supplement: Additional file 1 — Code and description to reproduce the figures in the manuscript. [file 12859_2015_454_MOESM1_ESM.pdf]

THE

The TagDust2 Benchmark

MANUAL

Timo Lassmann

11 Sepember 2014

Contents

Contents ii

1 Introduction 1

2 Figure 1: 3

3 Figure 2-5: Comparison to BTRIM, cutadapt and fastx_barcode_clipper. 5
- 3.1 Simreads 5
  - Simulation without linkers: 5
  - Simulation with linkers: 6
- 3.2 Notes on BTRIM, FastX and Cutadapt used in the benchmark 6
  - Program Versions: 6
- 3.3 Command line options used in the benchmark 6
  - TagDust 6
  - fastx_barcode_splitter.pl 7
  - Cutadapt 7
  - fastx_barcode_splitter.pl after cutadapt 7
  - Btrim 7
- 3.4 Evaluation 7
  - Notes on **evalres** 8
- 3.5 Plotting 8
  - File: plotting.R 8
- 3.6 File: run.mk 10
  - File: barread.sh 11
  - File: 5barread3.sh 14

4 Figure 6 19
- 4.1 File: process_umi_data.sh 19
- 4.2 File: make_umi_plots.R 23

5 Table 1: Comparison to Illumina's bcl2fastq. 25
- 5.1 Run the default pipeline on the validation data containing barcodes: 25
- 5.2 Run the pipeline without de-multiplecing and use TagDust2. 26
- 5.3 Run TagDust2 26
- 5.4 Comparison 27

6 Table 2: Summary of Single Cell extracted reads. 29

7 Additional files: 31
- 7.1 File: Figure1.tex: 31

Chapter 1

Introduction

The purpose of this document is to provide a complete description and the supporting code to reproduce the figures from the TagDust2 paper.

Typing **make** at the top level directory runs a C parser to extract bits of code embedded in this document. Among these is an additional file **run.mk**. Running:

```
bash-3.2$ make -f run.mk
```

will start re-running the benchmark to make Figures 1-5.

The other figures and table require downloading large datasets which are not included in this package.

The following chapters describe the code for Figures 1-6 and Table 1 and 2.

Chapter 2

Figure 1:

The figure is a drawing made in tikz [1]. The code is listed here.

To make the pdf version simply:

```
bash-3.2$ pdflatex Figure1.tex
```

[1] http://sourceforge.net/projects/pgf/

Chapter 3

Figure 2-5: Comparison to BTRIM, cutadapt and fastx_barcode_clipper.

The purpose to these benchmarks is to compare the accuracy and number of reads extracted by running different extraction programs.

All benchmarks are carried out using the following logic and programs

1) generate dataset using the program **simreads**
2) run program X,Y and Z
3) compare results using the program **evalres**

3.1 Simreads

SIMreads is a simple C program distributed alongside TagDust2 to simulate raw NGS reads, including barcodes, linkers and adaptors. The syntax is fairly specific to the types of benchmarks conducted in the TagDust2 paper but perhaps of some general use.

Apart from simulating the reads, *simreads* will also generate a number of auxiliary files needed by the de-multiplex programs used in the benchmark. These include:

- TagDust2 architecture files (*XXX_tagdust_arch.txt*)
- BTRIM pattern file (*XXX_btrim_pattern.txt*)
- FastX barcode file (*XXX_fastxbarcodefile.txt*)

Here are the command line options used:

Simulation without linkers:

Here I simulate NGS sequences containing a barcode at the 5' end followed by the read we wish to extract:

```
error=$(../../src/simreads $barcodes  -seed 42 -sim_barnum ${numbar[$c]}
   -sim_readlen 20 -sim_readlen_mod 0 -sim_numseq 100000 -sim_endloss 0 -
   sim_random_frac 0.1 -o $name   -sim_error_rate ${array[$j]}  2>&1 )
```

The important options include:

- **$barcodes** - points to a specific barcode file generated by the program design_edit_metric_tags.py from the EditTag package[1]. To ensure reproducibility I distribute TagDust2 with a selection of barcode files found in the dev/ directory.

[1]Faircloth BC, Glenn TC. 2012. Not all sequence tags are created equal: Designing and validating sequence identification tags robust to indels. PLoS ONE 7(8): e42543. doi: 10.1371/journal.pone.0042543

- **sim_error_rate** - the sequencer error rate. To generate the paper figures I vary the error rate from 1 to 3 percent in 0.5% increments.

- **sim_barnum** - specifies the number of barcodes used from the files pointed at by **$barcodes**.
- **sim_readlen** - specifies the length of the reads.
- **sim_numseq** - the number of sequences simulated.
- **sim_random_frac** - the fraction of sequences which are completely random. I.e. these should not be extracted.

Simulation with linkers:

Here I simulate NGS sequences as before but add 5' and 3' linkers:

```
error=$(../../src/simreads $barcodes  -sim_5seq agggaggacgatgcgg   -
   sim_3seq gtgtcagtcacttccagcgg  -seed 42 -sim_barnum ${numbar[$c]}  -
   sim_readlen 20 -sim_readlen_mod 0 -sim_numseq 100000 -sim_endloss 0 -
   sim_random_frac 0.1 -o $name   -sim_error_rate ${array[$j]}  2>&1 )
```

The important options include:

- **sim_5seq** - sequence appended to the 5' end (agggaggacgatgcgg)
- **sim_3seq** - sequence appended to the 3' end (gtgtcagtcacttccagcgg)

3.2 Notes on BTRIM, FastX and Cutadapt used in the benchmark

All programs usedfor the benchmark are distributed alongside TagDust2 distribution in /reproducibiliy/bin.

Program Versions:

BTRIM:

btrim64 - downloaded from:

http://graphics.med.yale.edu/trim/

btrim64 28-Jul-2014 15:56 35K

(version unknown)

cutadapt:

Version 1.3

fastx_barcode_splitter.pl

fastx_0.0.13

3.3 Command line options used in the benchmark

TagDust

TagDust is run in two different modes:

1) Basic mode - only one architecture is given.
2) Auto-detect mode - all architectures used in the **entire** benchmark are given and TagDust2 has to figure out which one to use.

Basic command

```
error=$( ../../src/tagdust -t 80  -seed 42  $name -arch $arch -o $outfile
    2>&1 )
```

- **$arch** - name of the file containing the architecture used in a particular test.
- **$name** - name of the input file.
- **$outfile** - name of the output file(s).

Auto-detect architecture

```
error=$( ../../src/tagdust -t 80  -seed 42  $name -arch all_arch.txt -o
    $outfile 2>&1 )
```

Here TagDust2 will search for the appropriate architecture in the file **all_arch.txt** no matter what the input is. Essentially this is how TagDust2 should be used in production environments. The user or operator specifies all the possible architectures based on the library preparation protocols used and TagDust2 automatically figures out which one is appropriate for each set of raw output files.

fastx_barcode_splitter.pl

```
error=$(  cat $name | ../bin/fastx_barcode_splitter.pl --bcfile $arch --
    prefix $outfile -bol 2>&1)
```

The $arch variable contains a link to the file containing the barcodes usedto simulate the sequences.

Cutadapt

Cutadapt is run first, then the output is de-multiplexed by fastx_barcode_splitter.pl.

```
error=$( ../bin/cutadapt --discard-untrimmed -n 2 --front agggaggacgatgcgg
         --adapter=gtgtcagtcacttccagcgg  $name   -o  $outfile  2>&1)
```

fastx_barcode_splitter.pl after cutadapt

Here is the command to de-multiplex the output of cutadapt.

```
error=$(  cat $cutadaptout | ../bin/fastx_barcode_splitter.pl --bcfile
    $arch --prefix $outfile -bol 2>&1)
```

Btrim

```
error=$( ../bin/btrim64 -p $arch  -t $name -o $outfile -l 18 -B  2>&1)
```

The file *$arch* contains the barcode sequences and the linkers.

3.4 Evaluation

During the simulation simreads adds the following labels to all reads:

1) **READ** or **RANDOM** - indicating whether the sequence is a random background sequence or not.
2) **SEQ** the actual read sequence before applying errors.
3) **RBC** the barcode sequence used for this read.
4) **BARNUM** the index of the barcode sequence.

Here is an example of a simulated read:

```
@READ0;SEQ:ACGGGGGTGCTTTCAATTGT;RBC:ACCA;BARNUM:7
agggaggacgatgcggACCAACGGGGGTGCTTTCAATTGTgtgtcagtcacttccagcgg
+
IIIIIIIIIIIIIIIIIIIIIIIIIIIIIIIIIIIIIIIIIIIIIIIIIIIIIIIIIIII
```

READ0 indicates that this is the first simulated sequence. The 7th barcode "*ACCA*" was used to index this read. Sequences in small caps are linkers added to either end.

The program **evalres**, part of the TagDust2 distribution, examines the output of the various programs and evaluates their performance.

Notes on evalres

The examination of the results works in the following ways:

1) firstly all output files are scanned. Each file labelled with the most frequently observed barcode in the file. If this results in an inconsistency, for example two files claiming to correspond to library 7 with the barcode "*ACCA*" the program quits with an error message.

2) Since we know the number of reads simulated and the fraction of *random* sequences we can compute the number of unextracted reads.

3) Calculating sensitivity and specificity. True positives (TP), false positives (FP), true negatives (TN) and false negatives (FN) are assigned in the following manner:

   - TP - number of reads in file **X** with barcode matching the file label (from step 1).
   - FP - number of reads in file **X** with barcodes not matching the file label. In other words these could be reads from one library assigned to another. In addition FP include random sequences wrongly assigned to a particular barcode.
   - TN - number of random sequences not present in any of of the labelled files.
   - FN - number of reads containing a barcode but absent from any of the labelled files.

3.5 Plotting

Here is the R code used to make figures 2-5.

File: plotting.R

```r

library(ggplot2)
library(gplots)
library(scales)
require("reshape")

yaxis_label_format <- function(x) {
  lab <- sprintf('%0.2f', x) # Format the strings as HH:MM:SS
}

mat = read.table("barread_4nt_4r.tsv",header =T,sep="\t")
mat$barcodes = paste(mat$barcodes, "Barcodes")
mat$barcodes  = factor(mat$barcodes, levels=c('8 Barcodes','24 Barcodes','
    48 Barcodes'))
s = subset(mat,mat$Program != "tagdustallarch")
s$Program  = factor(s$Program, levels=c('tagdust','fastx','btrim'))
m = melt(s, id=c("Program","simerror","barcodes"),measure.vars = c("Recall
    ", "Precision"))
```

```
ggplot(m, aes(x = simerror,y = value ,color=Program, fill=factor(Program))) + geom_line() +facet_grid(scales="free", variable ~ barcodes ) + scale_x_continuous(breaks=c(0.01,0.015,0.02,0.025,0.03), labels = expression("1","1.5","2","2.5","3")) + xlab("Simulated Error Rate (%)") + scale_y_continuous(labels=yaxis_label_format)+ ylab("")
ggsave("Figure2.pdf",width=8,height=4,dpi = 300)

mat = read.table("barread_6nt_4r.tsv",header =T,sep="\t")
mat$barcodes = paste(mat$barcodes, "Barcodes")
mat$barcodes = factor(mat$barcodes, levels=c('8 Barcodes','24 Barcodes','48 Barcodes'))
s = subset(mat,mat$Program != "tagdustallarch")
s$Program = factor(s$Program, levels=c('tagdust','fastx','btrim'))
m = melt(s, id=c("Program","simerror","barcodes"),measure.vars = c("Recall", "Precision"))
ggplot(m, aes(x = simerror,y = value ,color=Program, fill=factor(Program))) + geom_line() +facet_grid(scales="free", variable ~ barcodes ) + scale_x_continuous(breaks=c(0.01,0.015,0.02,0.025,0.03), labels = expression("1","1.5","2","2.5","3")) + xlab("Simulated Error Rate (%)") + scale_y_continuous(labels=yaxis_label_format)+ ylab("")
ggsave("Figure3.pdf",width=8,height=4,dpi = 300)

mat = read.table("5barread3_4nt_4r.tsv",header =T,sep="\t")
mat$barcodes = paste(mat$barcodes, "Barcodes")
mat$barcodes = factor(mat$barcodes, levels=c('8 Barcodes','24 Barcodes','48 Barcodes'))
s = subset(mat,mat$Program != "tagdustallarch")
s$Program = factor(s$Program, levels=c('tagdust','fastx','btrim'))
m = melt(s, id=c("Program","simerror","barcodes"),measure.vars = c("Recall", "Precision"))
ggplot(m, aes(x = simerror,y = value ,color=Program, fill=factor(Program))) + geom_line() +facet_grid(scales="free", variable ~ barcodes ) + scale_x_continuous(breaks=c(0.01,0.015,0.02,0.025,0.03), labels = expression("1","1.5","2","2.5","3")) + xlab("Simulated Error Rate (%)") + scale_y_continuous(labels=yaxis_label_format) + ylab("")
ggsave("Figure4.pdf",width=8,height=4,dpi = 300)


mat = read.table("5barread3_6nt_4r.tsv",header =T,sep="\t")
mat$barcodes = paste(mat$barcodes, "Barcodes")
mat$barcodes = factor(mat$barcodes, levels=c('8 Barcodes','24 Barcodes','48 Barcodes'))
s = subset(mat,mat$Program != "tagdustallarch")
s$Program = factor(s$Program, levels=c('tagdust','fastx','btrim'))
m = melt(s, id=c("Program","simerror","barcodes"),measure.vars = c("Recall", "Precision"))
ggplot(m, aes(x = simerror,y = value ,color=Program, fill=factor(Program))) + geom_line() +facet_grid(scales="free", variable ~ barcodes ) + scale_x_continuous(breaks=c(0.01,0.015,0.02,0.025,0.03), labels = expression("1","1.5","2","2.5","3")) + xlab("Simulated Error Rate (%)") + scale_y_continuous(labels=yaxis_label_format)+ ylab("")
ggsave("Figure5.pdf",width=8,height=4,dpi = 300)
```

3.6 File: run.mk

Runs all the scripts to make the paper figures 2-5. At first all the test datasets are generated once and their corresponding TagDust2 architecture files are collected. The file containing all architectures is then used to see whether TagDust2 can automatically detect the correct architectures when the actual benchmark is run later on.

```

.PHONY:  message benchmark rplot

benchmark: Figure1.pdf Figure2.pdf
	@echo Done

Figure1.pdf: Figure1.tex
	pdflatex $<;

Figure2.pdf: barread_6nt_4r.tsv barread_4nt_4r.tsv 5barread3_4nt_4r.tsv 5barread3_6nt_4r.tsv
	R --slave --vanilla < plotting.R
	rm Rplots.pdf


barread_4nt_4r.tsv: barread_results.txt
	cat barread_results.txt | grep _4nt_ | awk 'BEGIN{print "Program\tsimerror\tbarcodes\tRecall\tSpecificity\tPrecision\tKappa"} {if(NR>0){x = split($$1,a,"_");printf "%s\t%f\t%f\t%f\t%f\t%f\t%f\n" ,  a[1],a[2],a[3],$$2,$$3,$$4,$$5 }}' > barread_4nt_4r.tsv

5barread3_4nt_4r.tsv: 5barread3_results.txt
	cat 5barread3_results.txt |  grep _4nt_ | awk 'BEGIN{print "Program\tsimerror\tbarcodes\tRecall\tSpecificity\tPrecision\tKappa"} {if(NR>0){x = split($$1,a,"_");printf "%s\t%f\t%f\t%f\t%f\t%f\t%f\n" ,  a[1],a[2],a[3],$$2,$$3,$$4,$$5 }}' > 5barread3_4nt_4r.tsv

barread_6nt_4r.tsv: barread_results.txt
	cat barread_results.txt | grep _6nt_ | awk 'BEGIN{print "Program\tsimerror\tbarcodes\tRecall\tSpecificity\tPrecision\tKappa"} {if(NR>0){x = split($$1,a,"_");printf "%s\t%f\t%f\t%f\t%f\t%f\t%f\n" ,  a[1],a[2],a[3],$$2,$$3,$$4,$$5 }}' > barread_6nt_4r.tsv

5barread3_6nt_4r.tsv: 5barread3_results.txt
	cat 5barread3_results.txt |  grep _6nt_ | awk 'BEGIN{print "Program\tsimerror\tbarcodes\tRecall\tSpecificity\tPrecision\tKappa"} {if(NR>0){x = split($$1,a,"_");printf "%s\t%f\t%f\t%f\t%f\t%f\t%f\n" ,  a[1],a[2],a[3],$$2,$$3,$$4,$$5 }}' > 5barread3_6nt_4r.tsv

barread_results.txt: all_arch.txt
	./barread.sh -b ../../dev/EDITTAG_4nt_ed_2.txt -r
	./barread.sh -b ../../dev/EDITTAG_6nt_ed_3.txt -r

5barread3_results.txt: all_arch.txt
	./5barread3.sh -b ../../dev/EDITTAG_4nt_ed_2.txt -r
	./5barread3.sh -b ../../dev/EDITTAG_6nt_ed_3.txt -r

all_arch.txt:
```

```
    ./barread.sh -b ../../dev/EDITTAG_4nt_ed_2.txt
    ./barread.sh -b ../../dev/EDITTAG_6nt_ed_3.txt
    ./5barread3.sh -b ../../dev/EDITTAG_4nt_ed_2.txt
    ./5barread3.sh -b ../../dev/EDITTAG_6nt_ed_3.txt
    cat  *tagdust_arch.txt  | sort | uniq  > all_arch.txt

all: message

message:
    @echo To reproduce the figures run "make -f run.mk benchmark"
```

File: barread.sh

Runs a benchmark consiting of simulating sequences with a barcode followed by the reads. There are two options:

-b - should point to a file generated by *design_edit_metric_tags.py*

-r - toggle to run the benchmark. If not used, simreads will simulate reads and write TagDust architecture files but will not run the actual benchmark.

```
#!/bin/bash

echo "Running tagdust benchmarks:";

barcodes=
run=0

function usage()
{
cat <<EOF
usage: $0 -b  <barcode file> -r
EOF
exit 1;
}

while getopts b:r opt
do
case ${opt} in
b) barcodes=${OPTARG};;
r) run=1;;
*) usage;;
esac
done

if [ "${barcodes}" = "" ]; then usage; fi
```

Below are the ranges of error rates and number of barcodes used:

```
array=(0.01 0.015 0.02 0.025 0.03)
len=${#array[*]}

numbar=(8 24 48)
lenbar=${#numbar[*]}
```

If the benchmark should be run remove all temporary files left over from a previous run:

```
if [[ $run -eq 1 ]]; then
make --silent clean
fi

for (( c=0; c < $lenbar; c+=1 )); do
j=0

while [ $j -lt $len ]
do
name="barread${array[$j]}_${numbar[$c]}.fq"

## Simulation without linkers:

status=$?
if [[ $status -eq 0 ]]; then
printf "%20s%10s\n"  sim SUCCESS;
else
printf "%20s%10s\n"  sim FAILED;
printf "with ERROR $status and Message:\n\n$error\n\n";
exit 1;
fi

if [[ $run -eq 1 ]]; then

suffix="_tagdust_arch.txt"
arch=$name$suffix
suffix="_tagdust";
outfile=$name$suffix

## Basic command

status=$?
if [[ $status -eq 0 ]]; then
printf "%20s%10s\n"  tagdust SUCCESS;
else
printf "%20s%10s\n"  tagdust FAILED;
printf "with ERROR $status and Message:\n\n$error\n\n";
exit 1;
fi

suffix="_tagdustarchsel";
outfile=$name$suffix

## Auto-detect architecture
status=$?
if [[ $status -eq 0 ]]; then
printf "%20s%10s\n"  tagdust SUCCESS;
else
printf "%20s%10s\n"  tagdust FAILED;
printf "with ERROR $status and Message:\n\n$error\n\n";
exit 1;
fi

suffix="_fastxbarcodefile.txt"
arch=$name$suffix
```

```
suffix="_fastx";
outfile=$name$suffix

## fastx_barcode_splitter.pl
status=$?
if [[ $status -eq 0 ]]; then
printf "%20s%10s\n"  fastx_split SUCCESS;
else
printf "%20s%10s\n"  fastx_split FAILED;
printf "with ERROR $status and Message:\n\n$error\n\n";
exit 1;
fi

suffix="_tagdust_BC_";
outfile=$name$suffix

error=$( ../../src/evalres -name tagdust_${array[$j]}_${numbar[$c]}
    _$barcodes  $outfile*.fq -o barread  -sim_random_frac 0.1 -sim_numseq
    100000 2>&1)
status=$?
if [[ $status -eq 0 ]]; then
printf "%20s%10s\n"  eval_run SUCCESS;
else
printf "%20s%10s\n"  eval_run FAILED;
printf "with ERROR $status and Message:\n\n$error\n\n";
exit 1;
fi

suffix="_tagdustarchsel_BC_";
outfile=$name$suffix

error=$( ../../src/evalres -name tagdustallarch_${array[$j]}_${numbar[$c]}
    _$barcodes  $outfile*.fq -o barread  -sim_random_frac 0.1 -sim_numseq
    100000 2>&1)
status=$?
if [[ $status -eq 0 ]]; then
printf "%20s%10s\n"  eval_run SUCCESS;
else
printf "%20s%10s\n"  eval_run FAILED;
printf "with ERROR $status and Message:\n\n$error\n\n";
exit 1;
fi

suffix="_fastxBC";
outfile=$name$suffix

error=$( ../../src/evalres -name fastx_${array[$j]}_${numbar[$c]}
    _$barcodes $outfile* -o barread  -sim_random_frac 0.1 -sim_numseq
    100000 2>&1)
status=$?
if [[ $status -eq 0 ]]; then
printf "%20s%10s\n"  eval_run SUCCESS;
else
printf "%20s%10s\n"  eval_run FAILED;
printf "with ERROR $status and Message:\n\n$error\n\n";
```

```
exit 1;
fi
fi

let j++
let myrun++
done

done

if [[ $run -eq 1 ]]; then
make --silent clean
fi
```

File: 5barread3.sh

```
#!/bin/bash

echo "Running tagdust benchmarks:";

barcodes=
run=0

function usage()
{
cat <<EOF
usage: $0 -b  <barcode file> -r
EOF
exit 1;
}

while getopts b:r opt
do
case ${opt} in
b) barcodes=${OPTARG};;
r) run=1;;
*) usage;;
esac
done

if [ "${barcodes}" = "" ]; then usage; fi
```

Below are the ranges of error rates and number of barcodes used:

```

array=(0.01 0.015 0.02 0.025 0.03)
len=${#array[*]}

numbar=(8 24 48)
lenbar=${#numbar[*]}
```

If the benchmark should be run remove all temporary files left over from a previous run:

```

if [[ $run -eq 1 ]]; then
make --silent clean
```

```
fi

#barcodes="EDITTAG_4nt_ed_2.txt"

for (( c=0; c < $lenbar; c+=1 )); do

j=0

while [ $j -lt $len ]
do

name="5barread3${array[$j]}_${numbar[$c]}.fq"

## Simulation with linkers:

status=$?
if [[ $status -eq 0 ]]; then
printf "%20s%10s\n"  sim SUCCESS;
else
printf "%20s%10s\n"  sim FAILED;
printf "with ERROR $status and Message:\n\n$error\n\n";
exit 1;
fi

if [[ $run -eq 1 ]]; then
suffix="_tagdust_arch.txt"
arch=$name$suffix
suffix="_tagdust";
outfile=$name$suffix

## Basic command
status=$?
if [[ $status -eq 0 ]]; then
printf "%20s%10s\n"  tagdust SUCCESS;
else
printf "%20s%10s\n"  tagdust FAILED;
printf "with ERROR $status and Message:\n\n$error\n\n";
exit 1;
fi


suffix="_tagdustarchsel";
outfile=$name$suffix

## Auto-detect architecture
status=$?
if [[ $status -eq 0 ]]; then
printf "%20s%10s\n"  tagdust SUCCESS;
else
printf "%20s%10s\n"  tagdust FAILED;
printf "with ERROR $status and Message:\n\n$error\n\n";
exit 1;
fi

suffix="_btrim_pattern.txt"
```

```
arch=$name$suffix
suffix="_btrim";
outfile=$name$suffix

## Btrim
status=$?
if [[ $status -eq 0 ]]; then
printf "%20s%10s\n"  btrim SUCCESS;
else
printf "%20s%10s\n"  btrim FAILED;
printf "with ERROR $status and Message:\n\n$error\n\n";
exit 1;
fi

suffix="_cutadadapt.fq";
outfile=$name$suffix

## Cutadapt
status=$?
if [[ $status -eq 0 ]]; then
printf "%20s%10s\n"  cutadapt SUCCESS;
else
printf "%20s%10s\n"  cutadapt FAILED;
printf "with ERROR $status and Message:\n\n$error\n\n";
exit 1;
fi


suffix="_cutadadapt.fq";
cutadaptout=$name$suffix
suffix="_fastxbarcodefile.txt"
arch=$name$suffix
suffix="_fastx";
outfile=$name$suffix

## fastx_barcode_splitter.pl after cutadapt

status=$?
if [[ $status -eq 0 ]]; then
printf "%20s%10s\n"  fastx_split SUCCESS;
else
printf "%20s%10s\n"  fastx_split FAILED;
printf "with ERROR $status and Message:\n\n$error\n\n";
exit 1;
fi

suffix="_tagdust_BC_";
outfile=$name$suffix

error=$( ../../src/evalres -name tagdust_${array[$j]}_${numbar[$c]}
    _$barcodes  $outfile*.fq -o 5barread3  -sim_random_frac 0.1 -sim_numseq
     100000 2>&1)
status=$?
if [[ $status -eq 0 ]]; then
printf "%20s%10s\n"  eval_run SUCCESS;
```

```
else
printf "%20s%10s\n"  eval_run FAILED;
printf "with ERROR $status and Message:\n\n$error\n\n";
exit 1;
fi


suffix="_tagdustarchsel_BC_";
outfile=$name$suffix

error=$( ../../src/evalres -name tagdustallarch_${array[$j]}_${numbar[$c]}
    _$barcodes  $outfile*.fq -o 5barread3  -sim_random_frac 0.1 -sim_numseq
     100000 2>&1)
status=$?
if [[ $status -eq 0 ]]; then
printf "%20s%10s\n"  eval_run SUCCESS;
else
printf "%20s%10s\n"  eval_run FAILED;
printf "with ERROR $status and Message:\n\n$error\n\n";
exit 1;
fi

suffix="_btrim";
outfile=$name$suffix

error=$( ../../src/evalres -name btrim_${array[$j]}_${numbar[$c]}
    _$barcodes $outfile* -o 5barread3  -sim_random_frac 0.1 -sim_numseq
    100000 2>&1)
status=$?
if [[ $status -eq 0 ]]; then
printf "%20s%10s\n"  eval_run SUCCESS;
else
printf "%20s%10s\n"  eval_run FAILED;
printf "with ERROR $status and Message:\n\n$error\n\n";
exit 1;
fi

suffix="_fastxBC";
outfile=$name$suffix


error=$( ../../src/evalres -name fastx_${array[$j]}_${numbar[$c]}
    _$barcodes $outfile* -o 5barread3  -sim_random_frac 0.1 -sim_numseq
    100000 2>&1)
status=$?
if [[ $status -eq 0 ]]; then
printf "%20s%10s\n"  eval_run SUCCESS;
else
printf "%20s%10s\n"  eval_run FAILED;
printf "with ERROR $status and Message:\n\n$error\n\n";
exit 1;
fi

fi;

```

```
let j++
let myrun++
done

done

if [[ $run -eq 1 ]]; then
    make --silent clean
fi
```

Chapter 4

Figure 6

Here we test the ability of TagDust2 to work with complicated read architectures including fingerprints. We use the following raw data files from the paper "Counting absolute numbers of molecules using unique molecular identifiers." [1]:

- ERR048988.fastq.bz2
- ERR048990.fastq.bz2
- ERR048992.fastq.bz2
- ERR048994.fastq.bz2
- ERR048989.fastq.bz2
- ERR048991.fastq.bz2
- ERR048993.fastq.bz2

To extract the reads we use the following read architecture file:

```
bash-3.2$ cat arch.txt
tagdust -t 80 -1 F:NNN -2 S:T -3 F:NNNN -4 S:T -5 F:NNN -6 B:GACTT -7 S:
    GGGG -8 R:N -start 1 -end 25
```

indicating that we expect a 10 nucleotide fingerprint sequence (F:) separated by thymidines (S:) followed by a 5 nucleotide barcode (B:), 4 guanosines and finally the mappable read (R:).

The commands to process the fastq files are:

4.1 File: process_umi_data.sh

```
tagdust -arch arch.txt -t 80 ERR048988.fastq -o 15
    c_repeat1_lane1_extracted
tagdust -arch arch.txt -t 80 ERR048990.fastq -o 25
    c_repeat1_lane1_extracted
tagdust -arch arch.txt -t 80 ERR048992.fastq -o
    RNA_corresponding_to_1000_cells_lane1_extracted
tagdust -arch arch.txt -t 80 ERR048994.fastq -o
    RNA_corresponding_to_10_cells_lane1_extracted
tagdust -arch arch.txt -t 80 ERR048989.fastq -o 15
    c_repeat2_lane1_extracted
tagdust -arch arch.txt -t 80 ERR048991.fastq -o 25
    c_repeat1_lane2_extracted
```

[1] Teemu Kivioja, Anna VÃďhÃďrautio, Kasper Karlsson, Martin Bonke, Martin Enge, Sten Linnarsson, and Jussi Taipale. Counting absolute numbers of molecules using unique molecular identifiers. Nature methods, 9(1):72–74, January 2012.

```
tagdust -arch arch.txt -t 80 ERR048993.fastq -o
    RNA_corresponding_to_100_cells_lane1_extracted
```

Note that we rename the files to better understand which fastq file belongs to which experiment.

The next step is to map the sequences using bwa[2]:

```

for file in ./*
        do
                if [ -f $file ]; then
                        if [[ $file =~ _extracted_BC_GACTT.fq$ ]]; then
                                if [ -f $$file.sorted.bam ]; then
                                        echo "$file already processed"
                                else
                                                echo "working on $file"
                                                sed  -e 's/[ \t]//g' $file
                                                    > tmp.fq
                                                 bwa aln -t 80
                                                    $GENOME_DIR tmp.fq  |
                                                    bwa samse $GENOME_DIR
                                                    - tmp.fq | samtools
                                                    view -Su - | samtools
                                                    sort - $file.sorted

                                fi
                        fi
                fi
        done
```

$GENOME_DIR should point to a BWA index of the Drosophila melanogaster (BDGP5) gene sequences (downloaded from http://www.biomart.org).

Now we extract reads reads mapping to same location, and sort them by fingerprint ID, chromosome and position.

```
samtools view -F 16 -q 20 15c_repeat1_lane1_extracted_BC_GACTT.fq.sorted.
    bam | awk '{x = split($1,a,";"); b = substr(a[2],4);print b , $3 ,$4 }'
     | sort -S 16G -k 2,2 -k 3,3n -k 1,1n | uniq -c > 15
    c_repeat1_lane1_extracted_processed.csv

samtools view -F 16 -q 20
    RNA_corresponding_to_1000_cells_lane1_extracted_BC_GACTT.fq.sorted.bam
    | awk '{x = split($1,a,";"); b = substr(a[2],4);print b , $3 ,$4 }'|
    sort -S 16G -k 2,2 -k 3,3n -k 1,1n | uniq -c >
    RNA_corresponding_to_1000_cells_lane1_extracted_processed.csv


samtools view -F 16 -q 20 15c_repeat2_lane1_extracted_BC_GACTT.fq.sorted.
    bam | awk '{x = split($1,a,";"); b = substr(a[2],4);print b , $3 ,$4
    }'| sort -S 16G -k 2,2 -k 3,3n -k 1,1n | uniq -c > 15
    c_repeat2_lane1_extracted_processed.csv &

samtools view -F 16 -q 20
    RNA_corresponding_to_100_cells_lane1_extracted_BC_GACTT.fq.sorted.bam |
```

[2] Li H. and Durbin R. (2009) Fast and accurate short read alignment with Burrows-Wheeler Transform. Bioinformatics, 25:1754-60. [PMID: 19451168]

```
awk '{x = split($1,a,";"); b = substr(a[2],4);print b , $3 ,$4 }'| sort -S 16G -k 2,2 -k 3,3n -k 1,1n | uniq -c > RNA_corresponding_to_100_cells_lane1_extracted_processed.csv


samtools view -F 16 -q 20 25c_repeat1_lane1_extracted_BC_GACTT.fq.sorted.bam | awk '{x = split($1,a,";"); b = substr(a[2],4);print b , $3 ,$4 }' | sort -S 16G -k 2,2 -k 3,3n -k 1,1n | uniq -c > 25c_repeat1_lane1_extracted_processed.csv

samtools view -F 16 -q 20 RNA_corresponding_to_10_cells_lane1_extracted_BC_GACTT.fq.sorted.bam | awk  '{x = split($1,a,";"); b = substr(a[2],4);print b , $3 ,$4 }' | sort -S 16G -k 2,2 -k 3,3n -k 1,1n | uniq -c > RNA_corresponding_to_10_cells_lane1_extracted_processed.csv

samtools view -F 16 -q 20 25c_repeat1_lane2_extracted_BC_GACTT.fq.sorted.bam | awk  '{x = split($1,a,";"); b = substr(a[2],4);print b , $3 ,$4 }'  | sort -S 16G -k 2,2 -k 3,3n -k 1,1n | uniq -c > 25c_repeat1_lane2_extracted_processed.csv
```

Next we use the groupBy tool from the bedtool package [3] to either:

- sum or
- count

reads mapping to the same location with the same fingerprint.

```

cat 15c_repeat1_lane1_extracted_processed.csv | awk '{printf "%s\t%s\t%s\t%s\n",$1,$2,$3,$4}'  | groupBy  -g 3 -c 1 -o count > 15c_repeat1_lane1_UMI.csv
cat 15c_repeat1_lane1_extracted_processed.csv | awk '{printf "%s\t%s\t%s\t%s\n",$1,$2,$3,$4}'  | groupBy  -g 3 -c 1 -o sum > 15c_repeat1_lane1_NOUMI.csv

cat 15c_repeat2_lane1_extracted_processed.csv | awk '{printf "%s\t%s\t%s\t%s\n",$1,$2,$3,$4}'  | groupBy  -g 3 -c 1 -o count > 15c_repeat2_lane1_UMI.csv
cat 15c_repeat2_lane1_extracted_processed.csv | awk '{printf "%s\t%s\t%s\t%s\n",$1,$2,$3,$4}'  | groupBy  -g 3 -c 1 -o sum > 15c_repeat2_lane1_NOUMI.csv


cat 25c_repeat1_lane1_extracted_processed.csv | awk '{printf "%s\t%s\t%s\t%s\n",$1,$2,$3,$4}'  | groupBy  -g 3 -c 1 -o count > 25c_repeat1_lane1_UMI.csv
cat 25c_repeat1_lane1_extracted_processed.csv | awk '{printf "%s\t%s\t%s\t%s\n",$1,$2,$3,$4}'  | groupBy  -g 3 -c 1 -o sum > 25c_repeat1_lane1_NOUMI.csv

cat 25c_repeat1_lane2_extracted_processed.csv | awk '{printf "%s\t%s\t%s\t%s\n",$1,$2,$3,$4}'  | groupBy  -g 3 -c 1 -o count > 25c_repeat1_lane2_UMI.csv
```

[3] Quinlan, Aaron R., and Ira M. Hall. "BEDTools: a flexible suite of utilities for comparing genomic features." Bioinformatics 26.6 (2010): 841-842.

```
cat 25c_repeat1_lane2_extracted_processed.csv | awk '{printf "%s\t%s\t%s\t%s\n",$1,$2,$3,$4}'  | groupBy  -g 3 -c 1 -o sum > 25c_repeat1_lane2_NOUMI.csv


cat RNA_corresponding_to_1000_cells_lane1_extracted_processed.csv | awk '{printf "%s\t%s\t%s\t%s\n",$1,$2,$3,$4}'  | groupBy  -g 3 -c 1 -o count > RNA_corresponding_to_1000_cells_UMI.csv
cat RNA_corresponding_to_1000_cells_lane1_extracted_processed.csv | awk '{printf "%s\t%s\t%s\t%s\n",$1,$2,$3,$4}'  | groupBy  -g 3 -c 1 -o sum > RNA_corresponding_to_1000_cells_NOUMI.csv

cat RNA_corresponding_to_100_cells_lane1_extracted_processed.csv | awk '{printf "%s\t%s\t%s\t%s\n",$1,$2,$3,$4}'  | groupBy  -g 3 -c 1 -o count > RNA_corresponding_to_100_cells_UMI.csv
cat RNA_corresponding_to_100_cells_lane1_extracted_processed.csv | awk '{printf "%s\t%s\t%s\t%s\n",$1,$2,$3,$4}'  | groupBy  -g 3 -c 1 -o sum > RNA_corresponding_to_100_cells_NOUMI.csv

cat RNA_corresponding_to_10_cells_lane1_extracted_processed.csv | awk '{printf "%s\t%s\t%s\t%s\n",$1,$2,$3,$4}'  | groupBy  -g 3 -c 1 -o count > RNA_corresponding_to_10_cells_UMI.csv
cat RNA_corresponding_to_10_cells_lane1_extracted_processed.csv | awk '{printf "%s\t%s\t%s\t%s\n",$1,$2,$3,$4}'  | groupBy  -g 3 -c 1 -o sum > RNA_corresponding_to_10_cells_NOUMI.csv
```

Here we combine the results from replicas.

```

cat 15c_repeat1_lane1_NOUMI.csv 15c_repeat2_lane1_NOUMI.csv | sort -k 1,1 | groupBy  -g 1 -c 2 -o sum > 15c_combined_noumi.csv
cat 15c_repeat1_lane1_UMI.csv  15c_repeat2_lane1_UMI.csv  | sort -k 1,1  | groupBy  -g 1 -c 2 -o sum > 15c_combined_UMI.csv

cat 25c_repeat1_lane1_NOUMI.csv  25c_repeat1_lane2_NOUMI.csv | sort -k 1,1 |  groupBy  -g 1 -c 2 -o sum > 25c_combined_noumi.csv
cat 25c_repeat1_lane1_UMI.csv   25c_repeat1_lane2_UMI.csv | sort -k 1,1  | groupBy  -g 1 -c 2 -o sum > 25c_combined_UMI.csv
```

We now join the processed files either using UMIs or not for easy plotting with R:

```

join -a1 -a2 -1 1 -2 1 -o 0 1.2 2.2 -e "0" 15c_repeat1_lane1_UMI.csv  15c_repeat2_lane1_UMI.csv | awk '{printf "%s\t%d\t%d\n",$1,$2,$3 }' > comp_15c_umi.csv

join -a1 -a2 -1 1 -2 1 -o 0 1.2 2.2 -e "0" 25c_repeat1_lane1_NOUMI.csv  25c_repeat1_lane2_NOUMI.csv | awk '{printf "%s\t%d\t%d\n",$1,$2,$3 }' > comp_25c_noumi.csv

join -a1 -a2 -1 1 -2 1 -o 0 1.2 2.2 -e "0" 25c_repeat1_lane1_UMI.csv  25c_repeat1_lane2_UMI.csv | awk '{printf "%s\t%d\t%d\n",$1,$2,$3 }' > comp_25c_umi.csv

join -a1 -a2 -1 1 -2 1 -o 0 1.2 2.2 -e "0" 15c_repeat1_lane1_NOUMI.csv  25c_repeat1_lane1_NOUMI.csv | awk '{printf "%s\t%d\t%d\n",$1,$2,$3 }' >
```

```
comp_15c_repeat1_25c_repeat1_noumi.csv

join -a1 -a2 -1 1 -2 1 -o 0 1.2 2.2 -e "0" 15c_repeat1_lane1_UMI.csv  25c_repeat1_lane1_UMI.csv | awk '{printf "%s\t%d\t%d\n",$1,$2,$3 }' > comp_15c_repeat1_25c_repeat1_umi.csv

join -a1 -a2 -1 1 -2 1 -o 0 1.2 2.2 -e "0" 15c_repeat2_lane1_NOUMI.csv  25c_repeat1_lane2_NOUMI.csv | awk '{printf "%s\t%d\t%d\n",$1,$2,$3 }' > comp_15c_repeat2_25c_repeat1_lane2_noumi.csv

join -a1 -a2 -1 1 -2 1 -o 0 1.2 2.2 -e "0" 15c_repeat2_lane1_UMI.csv  25c_repeat1_lane2_UMI.csv | awk '{printf "%s\t%d\t%d\n",$1,$2,$3 }' > comp_15c_repeat2_25c_repeat1_lane2_umi.csv

join -a1 -a2 -1 1 -2 1 -o 0 1.2 2.2 -e "0" 15c_repeat1_lane1_NOUMI.csv  25c_repeat1_lane2_NOUMI.csv | awk '{printf "%s\t%d\t%d\n",$1,$2,$3 }' > comp_15c_repeat1_25c_repeat1_lane2_noumi.csv

join -a1 -a2 -1 1 -2 1 -o 0 1.2 2.2 -e "0" 15c_repeat1_lane1_UMI.csv  25c_repeat1_lane2_UMI.csv | awk '{printf "%s\t%d\t%d\n",$1,$2,$3 }' > comp_15c_repeat1_25c_repeat1_lane2_umi.csv

join -a1 -a2 -1 1 -2 1 -o 0 1.2 2.2 -e "0" 15c_repeat2_lane1_NOUMI.csv  25c_repeat1_lane1_NOUMI.csv | awk '{printf "%s\t%d\t%d\n",$1,$2,$3 }' > comp_15c_repeat2_25c_repeat1_lane1_noumi.csv

join -a1 -a2 -1 1 -2 1 -o 0 1.2 2.2 -e "0" 15c_repeat2_lane1_UMI.csv  25c_repeat1_lane1_UMI.csv | awk '{printf "%s\t%d\t%d\n",$1,$2,$3 }' > comp_15c_repeat2_25c_repeat1_lane1_umi.csv

join -a1 -a2 -1 1 -2 1 -o 0 1.2 2.2 -e "0"  15c_combined_noumi.csv 25c_combined_noumi.csv | awk '{printf "%s\t%d\t%d\n",$1,$2,$3 }' > 15_25_combined_noumi.csv
join -a1 -a2 -1 1 -2 1 -o 0 1.2 2.2 -e "0"  15c_combined_UMI.csv 25c_combined_UMI.csv | awk '{printf "%s\t%d\t%d\n",$1,$2,$3 }' > 15_25_combined_UMI.csv
```

4.2 File: make_umi_plots.R

Here are the commands used to generate panel one and two of Figure 6:

```
library(ggplot2)

mat = read.table("15_25_combined_noumi.csv",header = F);
cor = cor(mat[,2],mat[,3]);
frame=ggplot(mat,aes(mat[,2],mat[,3]))
frame + geom_point(colour="black",size=1,shape=20,alpha=0.3) + labs(x="15 cycles ",y="25 cycles",title="without UMI") + annotate("text", label = paste("R=", round (cor,6), sep=""), x = min(mat[,2])+10, y = max(mat[,3]) / 10, size = 6, colour = "red") +scale_x_log10() + scale_y_log10()+theme(axis.text=element_text(size=12), axis.title=element_text(size=14,face="bold"),title=element_text(size=18,face="bold"));
ggsave("15_25_combined_noumi.pdf")
mat = read.table("15_25_combined_UMI.csv",header = F);
```

```
cor = cor(mat[,2],mat[,3]);
frame=ggplot(mat,aes(mat[,2],mat[,3]))
frame + geom_point(colour="black",size=1,shape=20,alpha=0.3) + labs(x="15
   cycles",y="25 cycles",title="with UMI") + annotate("text", label =paste
   ("R=", round (cor,6), sep=""), x = min(mat[,2])+10, y = max(mat[,3]) /
   10, size = 6, colour = "red") +scale_x_log10() + scale_y_log10()+theme(
   axis.text=element_text(size=12), axis.title=element_text(size=14,face="
   bold"),title=element_text(size=18,face="bold"));
ggsave("15_25_combined_UMI.pdf")
```

Chapter 5

Table 1: Comparison to Illumina's bcl2fastq.

To run this benchmark some understanding on how to toggle the de-multiplexing options in the CASAVA pipeline are required. A great tutorial on this was written by Brant Faircloth[1].

It is necessary to install the bcl2fastq package[2] (bcl2fastq-1.8.4.tar.gz) and follow these steps:

5.1 Run the default pipeline on the validation data containing barcodes:

1) modify:

By default only one PhiX sample and one human sample are used for the validation. Modify:

```
./share/bcl2fastq-1.8.4/examples/Validation/110120_P20_0993_A805CKABXX/
   Data/Intensities/BaseCalls/SampleSheet.csv
```

NOTE: there are two validation datasets included but only 110120_P20_0993_A805CKABXX contains multiplexed samples.

so that all samples of lane 1 are de-multiplexed.

2) run:

```
bash-3.2$ configureValidation.pl \
        --source-dir ../share/bcl2fastq-1.8.4/examples/Validation/110120
           _P20_0993_A805CKABXX \
        --output-dir ./ValidationMultiplexed_casava
```

3) run base-caller with *default* de-multiplexing:

```
bash-3.2$ cd ValidationMultiplexed_casava
bash-3.2$ make
```

At this point we should have the CASAVA de-multiplexed fastq files.

[1] https://gist.github.com/brantfaircloth/3125885
[2] http://support.illumina.com/downloads/bcl2fastq_conversion_software_184.html

5.2 Run the pipeline without de-multiplecing and use TagDust2.

1) modify configuration makefile:

share/bcl2fastq-1.8.4/examples/Validation/110120_P20_0993_A805CKABXX/Unaligned/ValidationConfig.mk

from:

```
CONFIGURE_BCL_TO_FASTQ_PARAMS := --use-bases-mask 'y76n*,I6n,y76n*'
```

to:

```
CONFIGURE_BCL_TO_FASTQ_PARAMS := --use-bases-mask 'y76n*,Y6n,y76n*'
```

I.e. read two corresponding to the 6 nucleotide index is basecalled and written to a seperate file alongside the actual paired-end reads.

2) modify sample sheet:

/share/bcl2fastq-1.8.4/examples/Validation/110120_P20_0993_A805CKABXX/Data/Intensities/BaseCalls/SampleSheet.csv

to:

```
FCID,Lane,SampleID,SampleRef,Index,Description,Control,Recipe,Operator
A805CKABXX,1,not_demultiplexed,,,Test bcl conversion,N,D109LACXX,BCF,
   testbclconv
#A805CKABXX,1,AR005,human,ACAGTG,Cypress,Y,101+7,CB,Demo
#A805CKABXX,1,AR008,human,ACTTGA,Cypress,Y,101+7,CB,Demo
#A805CKABXX,1,PhiX,phix,TTAGGC,Cypress,Y,101+7,CB,Demo
#A805CKABXX,1,,unknown,Undetermined,Ignored clusters with unmatched
   barcodes for lane 1,N,101+7,CB,
```

Note that only lane 1 is included in the test.

3) run the pipeline again

```
bash-3.2$ configureValidation.pl \
         --source-dir ../share/bcl2fastq-1.8.4/examples/Validation/110120
            _P20_0993_A805CKABXX \
         --output-dir ./ValidationMultiplexed_casava
```

Now we have should have these three fastq files:

```
not_demultiplexed_NoIndex_L001_R1_001.fastq.gz
not_demultiplexed_NoIndex_L001_R2_001.fastq.gz
not_demultiplexed_NoIndex_L001_R3_001.fastq.gz
```

5.3 Run TagDust2

Here is the TagDust2 command:

```
bash-3.2$ tagdust -arch casava_arch.txt
   not_demultiplexed_NoIndex_L001_R1_001.fastq.gz
   not_demultiplexed_NoIndex_L001_R2_001.fastq.gz
   not_demultiplexed_NoIndex_L001_R3_001.fastq.gz  -t 80 -o demulti_large
```

Here are the contents of the architecture file casava_arch.txt:

```
tagdust -1 B:ACAGTG,ACTTGA,TTAGGC
tagdust -1 R:N
```

The first line corresponds to the architecture we wish to use on read number two containing the indices. The second line corresponds to *blank* architecture which results in the whole read to be extracted by TagDust2.

TagDust2 will:

1) scan each input file seperately with both architectures
2) determine that file:
    - one is a read (R:N)
    - two is contains indices (B:ACAGTG,ACTTGA,TTAGGC)
    - three is another read (R:N)
3) scan all reads with the *correct* architectures
4) extract reads in which the index could be unambigiously identified.

5.4 Comparison

To count the number of extracted reads (CASAVA):

```
bash-3.2$ zcat  Sample_AR005/AR005_ACAGTG_L001_R1_001.fastq.gz | wc -l
5890936

bash-3.2$ zcat  Sample_AR008/AR008_ACTTGA_L001_R1_001.fastq.gz  | wc -l
6072832

bash-3.2$ zcat Sample_PhiX/PhiX_TTAGGC_L001_R1_001.fastq.gz  | wc -l
196416
```

and the number of reads extracted by TagDust2:

```
bash-3.2$ wc  -l demulti_large_*READ1.fq
6035392 demulti_large_BC_ACAGTG_READ1.fq
6175672 demulti_large_BC_ACTTGA_READ1.fq
205952 demulti_large_BC_TTAGGC_READ1.fq
```

NOTE: the numbers above must be divided by 4 because each sequence corresponds to 4 lines in a fastq file.

To get an idea whether the reads additionally extracted by TagDust2 are of poor quality or not I aligned all reads to the human genome (hg38) / PhiX genome (NC 001422.1) using BWA-MEM[3]:

```
bash-3.2$ nice bwa  mem -t 16  /GROUP/REFERENCE/GRCh38/GRCh38.genome.fa
   demulti_large_BC_ACAGTG_READ1.fq demulti_large_BC_ACAGTG_READ2.fq   |
   samtools view -Sb - > tagdust_ACAGTG.bam

bash-3.2$ nice bwa  mem -t 16  /GROUP/REFERENCE/GRCh38/GRCh38.genome.fa
   demulti_large_BC_ACTTGA_READ1.fq demulti_large_BC_ACTTGA_READ2.fq   |
   samtools view -Sb - > tagdust_ACTTGA.bam
```

[3] Li, H.: Aligning sequence reads, clone sequences and assembly contigs with bwa-mem. arXiv preprint arXiv:1303.3997 (2013)

```
bash-3.2$ nice bwa  mem -t 16 phix.fa demulti_large_BC_TTAGGC_READ1.fq
   demulti_large_BC_TTAGGC_READ2.fq   | samtools view -Sb - >
   tagdust_TTAGGC.bam

bash-3.2$ nice bwa  mem -t 16  /GROUP/REFERENCE/GRCh38/GRCh38.genome.fa
   Sample_AR005/AR005_ACAGTG_L001_R1_001.fastq.gz Sample_AR005/
   AR005_ACAGTG_L001_R2_001.fastq.gz | samtools view -Sb - > casava_ACAGTG
   .bam

bash-3.2$ nice bwa  mem -t 16  /GROUP/REFERENCE/GRCh38/GRCh38.genome.fa
   Sample_AR008/AR008_ACTTGA_L001_R1_001.fastq.gz Sample_AR008/
   AR008_ACTTGA_L001_R2_001.fastq.gz | samtools view -Sb - > casava_ACTTGA
   .bam

bash-3.2$ nice bwa  mem -t 16 phix.fa Sample_PhiX/PhiX_TTAGGC_L001_R1_001.
   fastq.gz Sample_PhiX/PhiX_TTAGGC_L001_R2_001.fastq.gz  | samtools view
   -Sb - > casava_TTAGGC.bam
```

To count the number of correctly mapped and paired reads (excluding secondary and supplementary alignments):

```
bash-3.2$ samtools view -c -f 1 -F 2316 casava_ACAGTG.bam
bash-3.2$ samtools view -c -f 1 -F 2316  casava_ACTTGA.bam
bash-3.2$ samtools view -c -f 1 -F 2316 casava_TTAGGC.bam
bash-3.2$ samtools view -c -f 1 -F 2316 tagdust_ACAGTG.bam
bash-3.2$ samtools view -c -f 1 -F 2316 tagdust_ACTTGA.bam
bash-3.2$ samtools view -c -f 1 -F 2316 tagdust_TTAGGC.bam
```

Chapter 6

Table 2: Summary of Single Cell extracted reads.

Here we test how TagDust2 can handle datasets with a large number of indices / barcodes. We downloaded datafiles from [1]:

- Run00020_L3_1_110110_GA2X_00020_FC.fq.bz2
- Run00021_L3_1_110114_GA2X_00021_FC.fq.bz2
- Run00020_L5_1_110110_GA2X_00020_FC.fq.bz2
- Run00021_L6_1_110114_GA2X_00021_FC.fq.bz2
- Run00021_L1_1_110114_GA2X_00021_FC.fq.bz2

To extract the reads we use the following read architecture file:

```
bash-3.2$ cat arch.txt
tagdust -1 O:N -2 B:TTTAGG,ATTCCA,GCTCAA,CATCCC,TTGGAC,CTGTGT,GGACAT,
   CAAAGT,AAGCGG,AATAAA,GAGGAG,GGTACA,AGCGAG,GTCGGT,ATTTGC,AGGACT,GCCCTC,
   TCGTAA,CCAGAC,TATGTA,ACAATA,ATGCTT,AGTTTA,CACAAG,ATCAAC,TAGTCG,TAGAGA,
   GTCCCG,TACTTC,AAAGTT,TAAGGG,GTTGCC,AAGTAC,GATCTT,TTAACT,GCGAAT,CCGCTA,
   TGAAGC,ATACAG,CTTCTG,GAGATC,CCGACG,CTCCAT,AAAACG,TAGCAT,TCGGGT,GTGGTA,
   CCTAGA,GGGTTT,ATGGCG,TTCATA,AACGCC,GGCTGC,GCTGTG,AGATGG,GTAATG,AGGGTC,
   ATCTCT,GCCTAG,TCAAAG,CATGAT,TGTGCG,GCAGGA,TCTACC,AGTCGT,CGTGGC,GCGTCC,
   GAACGC,ACTTAT,TGGATG,TATTGT,ACGTTG,GAATTA,CCATCT,TGATCA,CGTATT,CGGCAG,
   GACACT,TTCCGC,CTCGCA,GTATAC,TGTCAC,TGCGGA,ACGAGC,ACACCC,CGCTTG,TGCAAT,
   CAACAA,CTGAAA,AACCTA,ACCTGA,TCACTT,GGGCGA,CGCACC,CGAGTA,CCTTTC -3 S:GGG
    -4 R:N -t 80
```

Here are the TagDust commands:

```
bash-3.2$ tagdust Run00020_L3_1_110110_GA2X_00020_FC.fq -arch arch.txt -t
   80 -o Run00020_L3_1_110110_GA2X_00020_FC_extracted.fq
bash-3.2$ tagdust Run00020_L5_1_110110_GA2X_00020_FC.fq -arch arch.txt -t
   80 -o Run00020_L5_1_110110_GA2X_00020_FC_extracted.fq
bash-3.2$ tagdust Run00021_L1_1_110114_GA2X_00021_FC.fq -arch arch.txt -t
   80 -o Run00021_L1_1_110114_GA2X_00021_FC_extracted.fq
bash-3.2$ tagdust Run00021_L3_1_110114_GA2X_00021_FC.fq -arch arch.txt -t
   80 -o Run00021_L3_1_110114_GA2X_00021_FC_extracted.fq
bash-3.2$ tagdust Run00021_L6_1_110114_GA2X_00021_FC.fq -arch arch.txt -t
   80 -o Run00021_L6_1_110114_GA2X_00021_FC_extracted.fq
```

[1] Islam, S., Kj ÌĹallquist, U., Moliner, A., Zajac, P., Fan, J.-B., L ÌĹonnerberg, P., Linnarsson, S.: Characterization of the single-cell transcriptional landscape by highly multiplex rna-seq. Genome research 21(7), 1160–1167 (2011)

All resulting reads were aligned to the mouse genome (GRCm38.p2) using a RNAseq pipeline. The latter is included in the TagDust package (./reproducibility/rnaseq_pipeline_4tagdust_paper-0.8.tar.gz).

Chapter 7

Additional files:

7.1 File: Figure1.tex:

```

\documentclass{article}
\usepackage[margin=0.2cm,  left=0.0cm, paperwidth=18.5cm, paperheight=17cm
   ]{geometry}
\usepackage{tikz}
\usetikzlibrary{shapes}
\usetikzlibrary{calc,backgrounds}
\usetikzlibrary{automata}
\usetikzlibrary{arrows,positioning,calc,matrix}
\usetikzlibrary{decorations.pathreplacing,shapes.multipart}

\tikzstyle{Dstate}=[shape=circle,draw=black!50,fill=black!10]
\tikzstyle{Istate}=[shape=diamond,draw=black!50,fill=black!10]
\tikzstyle{Mstate}=[shape=rectangle,draw=black!50,fill=black!10]

\tikzstyle{empty}=[shape=circle]

\tikzstyle{lightedge}=[->,dotted,thick]
\tikzstyle{mainstate}=[state,thick]
\tikzstyle{mainedge}=[->,thick]

%\renewcommand{\chaptername}{}
%\renewcommand{\thechapter}{}

\definecolor{black}{RGB}{0,0,0}
\definecolor{darkgrey}{RGB}{64,64,64}
\definecolor{grey}{RGB}{127,127,127}
\definecolor{lightgrey}{RGB}{230,230,230}

% scheme 1
\definecolor{winered}{RGB}{158,16,0}
\definecolor{lightred}{RGB}{199,53,42}
\definecolor{brown}{RGB}{158,95,0}
\definecolor{orange}{RGB}{235,141,0}

%scheme2

\definecolor{darkblue}{RGB}{19,48,182}
```

```
\definecolor{blue}{RGB}{36,89,158}
\definecolor{browngreen}{RGB}{82,75,19}
\definecolor{lightbrown}{RGB}{158,126,36}

%scheme3

\definecolor{green}{RGB}{52,132,23}


\definecolor{lightgreen}{RGB}{82,209,36}
\definecolor{lightbrowngreen}{RGB}{125,133,23}
\definecolor{yellowgreen}{RGB}{198,209,36}

%scheme4


\definecolor{paper}{RGB}{240,238,183}
\definecolor{organicgrey}{RGB}{163,162,124}


\definecolor{metal}{RGB}{75,81,82}
\begin{document}
\begin{tikzpicture}[]
 \tiny


 \node[shape=circle,draw=black,thick,font =\Large] at (-3,6.5) {\bf 1};
 \node[shape=circle,draw=black,thick,font =\Large] at (-3,0) {\bf 2};
  \node[shape=circle,draw=black,thick,font =\Large] at (-3,-6) {\bf 3};
   \node[shape=circle,draw=black,thick,font =\Large] at (-3,-8) {\bf 4};

   \node[style={align=center},draw = black, thick,font=\large] at (6,6.5) { \tt tagdust2 -1 O:N -2 B:GTA,AAC -3 R:N -4 L:CCTTAA  reads.fq} ;

      \path[shade,top color=gray!5,bottom color=blue!10!white] (2.2,6.2)--(3.2,6.2) -- (0.8,4.6) --  (-0.7,4.6) -- cycle;

   \path[shade,top color=gray!5,bottom color=blue!10!white] (4,6.2)--(6,6.2) -- (4.5,4.6) --  (1.6,4.6) -- cycle;

   \path[shade,top color=gray!5,bottom color=blue!10!white] (6.8,6.2)--(7.5,6.2) -- (6.7,4.6) --  (5.3,4.6) -- cycle;

   \path[shade,top color=gray!5,bottom color=blue!10!white] (8.3,6.2)--(10.2,6.2) -- (13.4,4.6) --  (7.5,4.6) -- cycle;



  \draw[
        -triangle 90,
        line width=2mm,
        postaction={draw, line width=0.5cm, shorten >=0.5cm, -}
    ] (6,6) -- node[right=1,font =\Large] {{\bf HMM construction}} (6,5) ;
```

```
\path[shade,top color=gray!5,bottom color=blue!10!white] (-0.65,-6.25)-- (12.25,-6.25) -- (12.5,-8.15) -- (-0.5,-8.25) -- (-0.65,-6.25);

\path[top color=winered!80!white,bottom color=winered!30!white] (-1.75,-6.25)-- (-0.65,-6.25) -- (6,-7.7) -- (2.5,-7.7) -- (-1.75,-6.25);

\draw[rounded corners=4pt,draw=lightgrey, fill=lightgrey] (-0.75,4.5) rectangle (0.75,-4);

\draw[rounded corners=4pt,draw=lightred, fill=lightred] (1.5,4.5) rectangle (4.5,-4);


\draw[rounded corners=4pt,draw=blue, fill=blue] (5.25,4.5)  rectangle (6.75,-4);

\draw[rounded corners=4pt,draw=lightgrey, fill=lightgrey] (7.5,4.5) rectangle (13.5,-4);

 \node  at (0,4)  [font=\Large,style={align=center}] () {{\bf Opt}};

 \node  at (3,4)  [font=\Large,style={align=center}] () {{\bf Barcode}};

%  \node  at (3,2+1.75)  [font=\Large,style={align=center}] () {{\bf 1}};

 %   \node  at (3,-2+ 1.75)  [font=\Large,style={align=center}] () {{\bf 2}};
  \node  at (6,4)  [font=\Large,style={align=center}] () {{\bf Read}};

    \node  at (10.5,4)  [font=\Large,style={align=center}] () {{\bf Linker}};

\draw[
        -triangle 90,
        line width=2mm,
        postaction={draw, line width=0.5cm, shorten >=0.5cm, -}
    ] (6,-4.5) -- node[right=1,font =\Large] {{\bf Decoding}} (6,-5.5) ;

  \node[draw=white, thick,font=\Large] at (6,-6)  {\bf {\color{lightgrey}C}{\color{lightred}GTA}{\color{blue}GGGGAACCCCGCCTGTTTACCAAAAACATCA}{\color{lightgrey}CCTTA}} ;

  \draw[
        -triangle 90,
        line width=2mm,
        postaction={draw, line width=0.5cm, shorten >=0.5cm, -}
    ] (6,-6.5) -- node[right=1,font =\Large] {{\bf Output}} (6,-7.5) ;

  \node[style={align=left},text width=27em, thick,font=\Large] at (2.8,-8) {\bf  $>$READ X, {\color{lightred}Barcode GTA}  } ;
```

```
    \node[style={align=left},text width=20em,draw=white, thick,font=\Large] at (2,-8.5)  {\bf   {\color{blue}GGGGAACCCCGCCTGTTTACCAAAAACATCA}};

\begin{scope}[shift={(-1.5,0)}];
\node[Dstate] (START) at (0,0){$S$};
\end{scope}

\begin{scope}[shift={(0,1.5)}];
\node[Mstate] (O1) at (0,0){\shortstack{ A\\  C\\  G\\ T} };
\end{scope}

\begin{scope}[shift={(2,2)}];
%\node[Dstate] (d1) at (0,1){$D$};
\node[Istate] (i1) at (0,0){$I$};
\node[Mstate] (m1) at (0,-1.2){\shortstack{ {\color{black!40}A}\\   {\color{black!40}C}\\  {\bf G}\\   {\color{black!40}T}} };

\node[Dstate] (d2) at (1,1){$D$};
\node[Istate] (i2) at (1,0){$I$};
\node[Mstate] (m2) at (1,-1.2){\shortstack{ {\color{black!40}A}\\   {\color{black!40}C}\\   {\color{black!40}G}\\   {\bf T}} };

%\node[Dstate] (d3) at (2,1){$D$};
%\node[Istate] (i3) at (2,-0){$I$};
\node[Mstate] (m3) at (2,-1.2){\shortstack{ {\bf A}\\   {\color{black!40}C}\\   {\color{black!40}G}\\   {\color{black!40}T}} };
\end{scope}

\begin{scope}[shift={(2,-2)}];

%\node[Dstate] (d4) at (0,1){$D$};
\node[Istate] (i4) at (0,0){$I$};
\node[Mstate] (m4) at (0,-1.2){\shortstack{ {\bf A}\\   {\color{black!40}C}\\   {\color{black!40}G}\\   {\color{black!40}T}} };

\node[Dstate] (d5) at (1,1){$D$};
\node[Istate] (i5) at (1,0){$I$};
\node[Mstate] (m5) at (1,-1.2){\shortstack{ {\bf A}\\   {\color{black!40}C}\\   {\color{black!40}G}\\   {\color{black!40}T}} };

%\node[Dstate] (d6) at (2,1){$D$};
%\node[Istate] (i6) at (2,-0){$I$};
\node[Mstate] (m6) at (2,-1.2){\shortstack{ {\color{black!40}A}\\   {\bf C}\\   {\color{black!40}G}\\   {\color{black!40}T}} };
\end{scope}
```

```


\begin{scope}[shift={(6,0)}];



\node[Mstate] (R1) at (0,0){\shortstack{ A\\  C \\ G \\   T} };
\end{scope}



\begin{scope}[shift={(8,2)}];

%CCTTAAGG
%\node[Dstate] (dl1) at (0,1){$D$};
\node[Istate] (il1) at (0,0){$I$};
\node[Mstate] (ml1) at (0,-1.2){\shortstack{ {\color{black!40}A}\\  {\bf C } \\   {\color{black!40}G}\\ {\color{black!40}T}} };



\node[Dstate] (dl2) at (1,1){$D$};
\node[Istate] (il2) at (1,0){$I$};
\node[Mstate] (ml2) at (1,-1.2){\shortstack{ {\color{black!40}A}\\   {\bf C}\\   {\color{black!40}G}\\   {\color{black!40}T}} };


\node[Dstate] (dl3) at (2,1){$D$};
\node[Istate] (il3) at (2,-0){$I$};
\node[Mstate] (ml3) at (2,-1.2){\shortstack{ {\color{black!40}A}\\   {\color{black!40}C}\\   {\color{black!40}G}\\   {\bf T}} };

\node[Dstate] (dl4) at (3,1){$D$};
\node[Istate] (il4) at (3,0){$I$};
\node[Mstate] (ml4) at (3,-1.2){\shortstack{ {\color{black!40}A}\\   {\color{black!40}C}\\   {\color{black!40}G}\\   {\bf T}} };

\node[Dstate] (dl5) at (4,1){$D$};
\node[Istate] (il5) at (4,0){$I$};
\node[Mstate] (ml5) at (4,-1.2){\shortstack{ {\bf A}\\   {\color{black!40}C}\\   {\color{black!40}G}\\   {\color{black!40}T}} };
%\node[Dstate] (dl6) at (5,1){$D$};
%\node[Istate] (il6) at (5,-0){$I$};
\node[Mstate] (ml6) at (5,-1.2){\shortstack{ {\bf A } \\   {\color{black!40}C}\\   {\color{black!40}G}\\   {\color{black!40}T}} };
\end{scope}

\begin{scope}[shift={(14,-2)}];
\node[Dstate] (END) at (0,0){$E$};
\end{scope}

 \path

(START) edge [lightedge] (O1)

```

```
(START) edge [lightedge] (m1)

(START) edge [lightedge] (m4)

(O1) edge [lightedge, loop above] ()
(R1) edge [lightedge, loop above] ()

(O1)edge [lightedge] (m1)
(O1)edge [lightedge] (m4)
(R1)edge [lightedge] (ml1)

(ml1.south)edge [lightedge] (END)
(ml2.south)edge [lightedge] (END)
(ml3.south)edge [lightedge] (END)
(ml4.south)edge [lightedge] (END)
(ml5.south)edge [lightedge] (END)
(ml6.south)edge [lightedge] (END)
(R1) edge [lightedge](END)

(m3)edge [lightedge] (R1)
(m6)edge [lightedge] (R1)

(m1) edge [lightedge] (m2)
(m1) edge [lightedge] (d2)
(m1) edge [lightedge] (i1)

(i1) edge [lightedge,loop above] ()
(i1) edge  [lightedge] (m2)
(i1) edge [lightedge, loop above] ()
%(d1) edge  [lightedge,->] (d2)
%(d1) edge  [lightedge,->] (m2)

(m2) edge [lightedge] (m3)
%(m2) edge [lightedge] (d3)
(m2) edge [lightedge] (i2)

(i2) edge [lightedge,loop above] ()
(i2) edge  [lightedge] (m3)
(i2) edge [lightedge, loop above] ()
%(d2) edge  [lightedge,->] (d3)
(d2) edge  [lightedge,->] (m3)


%(m3) edge [lightedge] (i3)

%(i3) edge [lightedge,loop above] ()



(m4) edge [lightedge] (m5)
(m4) edge [lightedge] (d5)
(m4) edge [lightedge] (i4)

(i4) edge [lightedge,loop above] ()
(i4) edge  [lightedge] (m5)
```

```
(i4) edge [lightedge, loop above] ()
%(d4) edge  [lightedge,->] (d5)
%(d4) edge  [lightedge,->] (m5)

(m5) edge [lightedge] (m6)
%(m5) edge [lightedge] (d6)
(m5) edge [lightedge] (i5)

(i5) edge [lightedge,loop above] ()
(i5) edge  [lightedge] (m6)
(i5) edge [lightedge, loop above] ()
%(d5) edge  [lightedge,->] (d6)
(d5) edge  [lightedge,->] (m6)


%(m6) edge [lightedge] (i6)

%(i6) edge [lightedge,loop above] ()



(ml1) edge [lightedge] (ml2)
(ml1) edge [lightedge] (dl2)
(ml1) edge [lightedge] (il1)

(il1) edge [lightedge,loop above] ()
(il1) edge  [lightedge] (ml2)
(il1) edge [lightedge, loop above] ()
%(dl1) edge  [lightedge,->] (dl2)
%(dl1) edge  [lightedge,->] (ml2)

(ml2) edge [lightedge] (ml3)
(ml2) edge [lightedge] (dl3)
(ml2) edge [lightedge] (il2)

(il2) edge [lightedge,loop above] ()
(il2) edge  [lightedge] (ml3)
(il2) edge [lightedge, loop above] ()
(dl2) edge  [lightedge,->] (dl3)
(dl2) edge  [lightedge,->] (ml3)


(ml3) edge [lightedge] (ml4)
(ml3) edge [lightedge] (dl4)
(ml3) edge [lightedge] (il3)

(il3) edge [lightedge,loop above] ()
(il3) edge  [lightedge] (ml4)
(il3) edge [lightedge, loop above] ()
(dl3) edge  [lightedge,->] (dl4)
(dl3) edge  [lightedge,->] (ml4)


(ml4) edge [lightedge] (ml5)
(ml4) edge [lightedge] (dl5)
```

```
(ml4) edge [lightedge] (il4)

(il4) edge [lightedge,loop above] ()
(il4) edge  [lightedge] (ml5)
(il4) edge [lightedge, loop above] ()
(dl4) edge  [lightedge,->] (dl5)
(dl4) edge  [lightedge,->] (ml5)



(ml5) edge [lightedge] (ml6)
%(ml5) edge [lightedge] (dl6)
(ml5) edge [lightedge] (il5)

(il5) edge [lightedge,loop above] ()
(il5) edge  [lightedge] (ml6)
(il5) edge [lightedge, loop above] ()
%(dl5) edge  [lightedge,->] (dl6)
(dl5) edge  [lightedge,->] (ml6)
;

\end{tikzpicture}

\end{document}
```
